# Serotyping of Actinobacillus pleuropneumoniae based on whole genome sequencing: validation of a bioinformatic tool

**DOI:** 10.1099/mgen.0.001434

**Published:** 2025-07-15

**Authors:** Øystein Angen, Kasper Thystrup Karstensen, Anna Vilaró, Lina Maria Cavaco, Paul R. Langford, Lorenzo Jose Fraile Sauce, Lourdes Migura-Garcia, Charlotte Mark Salomonsen, Yanwen Li, Janine T. Bossé

**Affiliations:** 1 Department of Bacteria, Parasites, and Fungi, Statens Serum Institut (SSI), Copenhagen, Denmark; 2 Grup de Sanejament Porcí (GSP), Lleida, Spain; 3 Section of Paediatric Infectious Disease, Department of Infectious Disease, Imperial College London, London, UK; 4 Department of Animal Science, ETSEA, Universitat de Lleida-AGROTECNIO-CERCA Centre, Lleida, Spain; 5 Institute of Agrifood Research and Technology (IRTA-CReSA), Animal Health Program (CReSA), Collaborating Centre of the World Organization for Animal Health for Research and Control of Emerging and Re-emerging Pig Diseases in Europe, Campus de la Universitat Autònoma de Barcelona (UAB), Barcelona, Spain; 6 Veterinary Laboratory, Danish Agriculture and Food Council, Kjellerup, Denmark

**Keywords:** *Actinobacillus pleuropneumoniae*, serotyping, whole genome sequencing (WGS)

## Abstract

Serovar detector is a new bioinformatic tool for determining the serovar of *Actinobacillus pleuropneumoniae* using whole genome sequencing. The composition of *cps* genes of isolates is compared to those of the serovar reference strains, and the outcome is determined both by the number of common genes and the similarities between the homologous genes. The validation of the bioinformatic tool utilized a broad collection of 732 isolates, including representatives from all described serovars. The isolates included had been characterized by conventional serotyping, PCR tests or different bioinformatic tools. The collection also includes isolates that have been difficult to allocate to a serovar using serology to test the performance of the Serovar detector when potential new varieties or combinations of *cps* genes are present. Out of the 732 isolates included in the investigation, only 36 isolates (4.9%) could not be allocated to the 19 recognized serovars. The validation showed that the Serovar detector is a robust method for determining the serovar of an isolate and a valuable tool for further characterization of the genetic heterogeneity both within serovars and within the *A. pleuropneumoniae* species.

## Data Summary

WGS data for the isolates have been deposited in the NCBI Sequence Read Archive under the BioProject PRJEB85426, and the accession numbers can be found in a supplementary table. The collection also includes a subset of Spanish isolates which have earlier been deposited on NCBI under the BioProjects PRJNA1062770 and PRJNA781224, as well as Norwegian and Danish isolates which have been deposited under the BioProject PRJEB47034.

Serovar detector is freely available at https://github.com/KasperThystrup/serovar_detector.

**Impact Statement**

*Actinobacillus pleuropneumoniae* is an important cause of pneumonia and mortality in pigs worldwide. Serotyping is important for describing variation among strains and to indicate virulence. Serovar detector is an important contribution to the field by describing a method for determining the serovar of an isolate based on whole genome sequencing. The investigation shows that the Serovar detector is a robust method for allocating unknown isolates to a serovar in a quick and reproducible manner. Additionally, the method will supply information on variation in the detected capsular polysaccharide synthesis genes that might give rise to further investigations and possibly descriptions of additional serovars.

## Introduction

*Actinobacillus pleuropneumoniae* is a Gram-negative, encapsulated respiratory pathogen of swine and the causative agent of porcine pleuropneumonia. The disease occurs worldwide and causes high mortality and large economic losses to the swine industry [1,2]. So far, 19 different serovars of *A. pleuropneumoniae* have been described [3,4]. The species can furthermore be divided into two biovars based on the requirement for NAD for growth (biovar 1 being NAD-dependent). Differentiation between serovars is predominately based on structural differences in the capsular polysaccharides (CPSs) and, for most serovars, also differences in lipopolysaccharides [5,6]. In some strains, unusual patterns of surface antigens can occur, e.g. in those having a CPS similar to that of serovar 2 and a lipopolysaccharide structure similar to that of serovar 7, which were designated K2:O7 [7]. Some of these isolates were later allocated to the new serovars 17 and 18 [8]. Isolates with other composite serotypes such as K1:O7, K2:O4, K6b:O3 and K12:O3 have also been described [9,13].

Studies have indicated considerable differences in virulence between serovars, partly due to the different combinations of toxins produced by each serovar [14,17]. Serotyping has been a commonly used technique for epidemiological monitoring of the disease in swine herds and for subsequent decisions on herd health status, prevention, therapy and eradication. Many countries have long-term programmes for targeting specific serovars of *A. pleuropneumoniae*. In Denmark, serological monitoring is a central part of the health classification in the specific-pathogen-free system [18]. In a geographically restricted area, usually a few serovars dominate. In North America, serovars 1, 5 and 7 are the most commonly isolated, whereas in Germany, serovars 2, 7 and 9 are the most prevalent [19]. In Korea, serovars 2, 5 and 6 predominate [20], whereas in the United Kingdom, serovar 8 predominates [21]. In Spain, serovars 9/11, 4 and 17 are the most commonly isolated [17]. In Denmark, serovars 2, 6 and 12 account for ~94% of the strains isolated from swine with clinical diseases [22]. However, due to the restricted numbers of serovars that tend to dominate in different regions, serotyping often fails to provide sufficient information for tracing infections.

The ability to discriminate between serovars is nevertheless very important, as there are differences in geographical distribution that are not static [1,6], as well as differences in levels of virulence [2,23]. Thus, accurate typing is essential for diagnosis and for tracking the emergence of serovars rarely, or not previously, reported within a geographical region.

Although a number of serological tests are available for typing *A. pleuropneumoniae* isolates [6], the need for high-quality reference antisera limits the number of laboratories able to perform diagnostics, and even then, problems with cross-reactivity between certain serovars are unavoidable. Increasingly, laboratories are using molecular typing methods to more accurately and reproducibly identify *A. pleuropneumoniae* isolates [1,6]. During the last decades, a number of PCR assays have been described, many of which were based on incomplete sequences of the CPS biosynthetic loci. Whole genome sequencing (WGS) has given the opportunity for a more detailed characterization of the *cps* operons and a more optimal design of serotype-specific primers. PCR assays have now been developed for the detection of specific *cps* genes in all the currently recognized 19 serovars [3,4].

With the increased availability of WGS, a number of diagnostic laboratories now utilize this technology for obtaining a more specific identification and characterization of bacterial isolates. WGS opens up opportunities for epidemiological tracing and comparison of isolates on both a national and global scale and offers a more detailed characterization of the *cps* operon. Currently, serotyping from WGS data can be performed manually by identifying the *cps* genes and comparing these to the *cps* operons of the serovar reference strains. This can ensure an accurate determination of the serovar, although the process can be quite tedious and time-consuming.

In this article, we describe a reliable *in silico* method for performing serotyping from WGS data from an isolate. The method which has been developed into a bioinformatic tool called Serovar detector automates the process of detecting and comparing the composition of *cps* genes against the serovar reference strains. It supports input from both raw read sequencing data and WGS assemblies. The output of the Serovar detector is a summary of the detected *cps* genes as well as a suggestion of the serovar for each of the included isolates. The suggestion is based on the quantity and composition of *cps* genes. Finally, the tool was implemented into a Snakemake framework to support pipeline functionality for large isolate collections. Serovar detector is freely available at https://github.com/KasperThystrup/serovar_detector.

The validation of the method utilizes a broad geographical collection of isolates, including representatives from all described serovars. The isolates included have earlier been characterized by conventional serotyping, PCR tests or different bioinformatic tools. It also includes isolates that have been difficult to allocate to a serovar using serology in order to test the performance of the method when potential new varieties or combinations of *cps* genes are present. The validation shows that the Serovar detector represents a robust method for the determination of serovars and also a valuable tool for further characterization of the genetic heterogeneity both within the serovars and within the * A. pleuropneumoniae* species.

## Methods

### Bacterial isolates

Closed genomes were available for the reference strains of serovars 1–18 [24]. For serovar 19, genome sequences of the two reference strains from Denmark and Canada published by Stringer *et al*. [4] were included – as well as two published serovar 19 sequences from Switzerland [25].

From 2021, all Danish isolates of *A. pleuropneumoniae* that were isolated at the Veterinary Diagnostic Laboratory in Kjellerup (Danish Agriculture and Food Council) have been sent to Statens Serum Institut for WGS-based serotyping. In the years 2021–2023, a total of 308 isolates were serotyped based on WGS. In 2021, 119 of the isolates were also serotyped by slide agglutination at the Danish Veterinary Institute (Technical University of Denmark).

A collection of 169 Spanish isolates was included in the validation [17]. For these Spanish isolates, the serotype had been determined by a multiplex PCR [4] at Grup de Sanejament Porcí, Lleida, Spain.

A collection of serovar 8 isolates from Norway, Denmark and the UK was also included in the project [26]. In order to obtain a sufficient number of isolates from the different serovars, 37 isolates from the archives of DTU-VET were included, as well as 52 WGS sequences from the collection at Imperial College London.

A total of 732 WGS sequences from different serovars of *A. pleuropneumoniae* were included in the investigation. The distribution of serovars and the origin of strains can be found in Table 1.

**Table 1.** Strains included in validation of Serovar detector

Serovar*	Closed genomes*^a^*	Denmark	Spain^*b*^	Norway	UK	Other countries	Total
**1**	1	5				7 (3 China, 2 Cyprus, 2 Canada)	13
**2**	1	188	8	1		1 (Czechia)	199
**3**	1				2	2 (Switzerland, Germany)	5
**4**	1		29				30
**5**	2	5	18		1	2 (China, Cyprus)	28
**6**	1	59	3		3		66
**7**	1	8	1	2	3		15
**8**	1	28^*c*^	4	120^*c*^	19^*c*^		172
**9**†	1					2 (Germany)	3
**10**	1	4	2	1	1	1 (Germany)	10
**11**†	1					3 (France, Czechia, Cyprus)	4
**9/11**†			56				56
**12**	1	23		1	3		28
**13**	1		16				17
**14**	1	3				1 (Estonia)	5
**15**	1	1				2 (China)	4
**16**	1					4 (Hungary)	5
**17**	1	7	20			1 (Canada)	29
**18**	1	6					7
**19**		2				3 (2 Switzerland^*d*^, 1 Canada)	5
**K2:O7**		9	13			1 (France)	23
**NT**		6	1	1			8
Total	19	354	171	126	32	30	732

*Serovar indicates the output from the pipeline. *a*: Donà *et al.*, 2021 [24]; *b*: Vilaró *et al.* [17]; *c*: Cohen *et al.* [26].

†Strains received as serovar 9, 11 or 9/11 were all typed as serovar 11 by Serovar detector. *d*: Peterhans *et al.* [25].

nt, non-typeable

### Whole genome sequencing

Genomic DNA was extracted from the Danish and Norwegian isolates using the DNeasy Blood and Tissue Kit (QIAGEN, Germantown, MD, USA) and quantified on a Qubit 3.0 Fluorometer (Invitrogen, Waltham, MA, USA). Preparation of the DNA sequence libraries was performed using the Illumina Nextera XT DNA Library Preparation Kit (Illumina Inc., San Diego, CA, USA) and sequenced on a NextSeq 500 platform (Illumina Inc., San Diego, CA, USA) with paired-end sequencing (2×151 bp) using a 300-cycle NextSeq Mid-Output Kit followed by quality assessment using bifrost (https://github.com/ssi-dk/bifrost). The reads were *de novo* assembled using the SKESA assembler v. 2.2 [27] using default parameters.

Genomic sequences from clinical isolates from the UK and other countries (see Table 1) have been included from the archives of Imperial College London and were determined as described in Bossé *et al*. [28]. Genomic sequences from Spanish strains were obtained as described by Vilaró *et al*. [17].

For phylogenetic reconstruction, the sequence reads from all isolates were included. Using the closed chromosomal sequence of serovar 2 strain S1536 (GenBank accession number CP031875) as reference, after removal of duplicated regions using NUCmer v.3.1 [29], identification of SNPs was performed with NASP v.1.2.0 [30]. All positions with <10-fold sequencing depth and 90% unambiguous variant calls for any isolate were excluded. The removal of high-density SNP regions, such as those caused by recombination, was identified and removed using Gubbins v.2.3.4 [31] prior to phylogenetic reconstruction using FastTree v.2.1.8 [32].

Visualization of the phylogeny and metadata was performed using iTol v.6 (https://itol.embl.de).

### Reference strains

Reference genomes for each of the 19 determined serovars of *A. pleuropneumoniae* were downloaded from National Center for Biotechnology Information (NCBI). CPS biosynthesis gene sequences (*cps* genes), which are found in all *Actinobacillus* species between *modF* and *ydeN*, were extracted from the reference strains [3] (Table S1, available in the online Supplementary Material) and blasted (blastn) against each other to determine potential duplicate genes across multiple serovars (Table S2).

Since the composition of *cps* gene sequences differs between the serovars, each *cps* gene was named according to the serovar of their reference strain, i.e. *cpsB* genes of serovar 2 were named *cps2B*. Some *cps* sequences were completely identical throughout multiple serovars. These genes were named according to the lowest numerical serovar they were extracted from; e.g. *cpsB* in serovars 2, 6, 8, 13 and 17 are completely identical; thus, the gene was named *cps2B* for all serovars.

Each unique *cps* gene was collected and indexed using Kmer-aligner v.1.4.9 [33], hence referred to as the reference database. The composition of *cps* genes for each of the reference serovar strains was recorded as serovar profiles and used downstream for distinguishing serovars.

### Gene detection and serovar designation with Serovar detector

Isolate raw read sequencing data and assembly data were mapped against the reference database using Kmer-aligner. Next, the *cps* genes were compared against each serovar profile. Here, *cps* gene matches against each of the serovar profiles were counted, and the serovar profile with the highest total match count was designated the serovar of the isolate. Whenever a detected *cps* gene occurred throughout multiple serovar profiles, such as *cps2B* occurring in serovars 2, 6, 8, 13 and 17, the single *cps* gene resulted in a count for each of the corresponding serovar profiles. In case of multiple serovar profiles having match counts corresponding to the highest match count, the isolate was determined to be non-typeable.

To determine whether a composite serovar was recognizable through this approach, a serovar profile for K2:O7 was added along the profiles for the 19 described serovars (Table S1). The serovar profile was annotated according to the curated composition of *cps* genes as reported by Bosse *et al*. [3].

Mapping quality was assessed by splitting *cps* gene mappings into two subcategories: full matches with a coverage and identity of at least 98% and partial matches with a coverage and identity <98%. In order to assess the quality of the designated serovar, the full matches to the corresponding serovar profile were compared to the total amount of *cps* genes in the given serovar profile. If fewer than 50% of the isolate’s *cps* genes were full matches, the isolate was designated non-typeable. All these tasks were collected into a single bioinformatic tool which we named Serovar detector.

### Further characterization of non-typeable isolates

Isolates that did not yield conclusive results with the Serovar detector were further investigated by comparing the *cps* operons obtained by WGS to the different serovar reference strains using CLC (CLC Genomics Workbench, v. 20.0, Qiagen) or Geneious Prime (v. 2022.1.1).

## Results

### Output of Serovar detector

Out of the 732 isolates included in the investigation, only 36 isolates (4.9%) could not be allocated to one of the 19 recognized serovars (5 isolates as 9/11, 23 isolates as K2:O7 and 8 isolates as non-typeable). Out of the 696 typable isolates, 434 (62.4%) contained all the genes found in their respective serovar profile with a similarity above 98%. The biggest difference from this was observed within serovar 8, where 137 out of 172 isolates (79.7%) only contained full matches for seven out of the expected eight *cps* genes. In all these strains, *cps17D* was detected instead of *cps8D*. However, the similarity between these two genes is 99.93%, as there are only two mismatches between the genes. The Serovar detector output also shows which *cps* genes are detected with <98% identity and coverage as well as *cps* genes specific to other serovars. The output indicates the identity and the coverage of all detected genes (showing identities and coverages below 100%). The results from the Serovar detector on all 732 isolates are summarized in Table 2.

**Table 2.** Summary of the results from Serovar detector

Serovar	No.	Frequency*	No.	%	Serovar match†	Other genes (% similarity, min–max)‡
**1**	13	4 of 43 of 4	85	61.538.5	1A, 1B, 1C, 1D1B, 1C, 1D	19D (19.9–20.7)1A (56.6–90.1), 4A (82.3–90.8), 14A (38.4–87.8), 19A (29.8–77.0), 19D (19.4–20.4)
**2**	199	7 of 76 of 75 of 74 of 7	1662661	831330.5	2A, 2B, 2C, 2D, 2E, 2F, 2G2A, 2B, 2C, (2D), (2E), 2G2A, 2B, 2C, 2D, 2G2A, 2B, 2C, 2G	7B (76.9–97.8)2D (91.6–97.4), 2E (98), 2F (94.4–97.1)2E (87.6–97.0), 2F (96.4–97.6)2D (97.9), 2E (83.1), 2F (91.8)
**3**	5	7 of 7	5	100	3A, 3B, 3C, 3D, 3E, 3F, 3G	9D (7.6–9.9)
**4**	30	3 of 3	5	100	4A, 4B, 4C	1A (41.9–62.4), 18A (30.6–37.1)
**5**	28	3 of 32 of 3	271	96.43.6	5A, 5B, 5C5A, 5B	–5C (96.2)
**6**	66	7 of 76 of 75 of 74 of 7	56352	84.84.57.63.0	6A, 2B, 6C, 6D, 6E, 6F, 6G6A, 2B, 6C, 6D, (6E), 6F, (6G)6C, 6D, (6E), (6F), 6G6D, 6E, 6F, 6G	–6E (96.3–96.8), 2G (91.5)8A (86.4-90.0), 7A (46.2–76.4), 6A (50.7–59.9), 2B (99.8), 6E (97.3), 6G (95.5)8A (88.2–86.0), 7A (56.1–66.8), 6A (50.7–57.7), 2B (99.8), 6C (91.8–97.5), 2C (82.7–81.7)
**7**	15	5 of 54 of 5	132	86.713.3	7A, 7B, 7C, 7D, 7E7A, 7B, 7C, (7D), (7E)	–7D (97.1), 7E (95.9)
**8**	172	8 of 87 of 86 of 85 of 8	23137102	13.479.75.81.2	8A, 2B, 8C, 8D, 8E, 8F, 8G, 8H8A, 2B, 8C, 8E, 8F, 8G, (8H)8A, 2B, 8C, 8E, (8F), (8G), (8H)8A, 2B, 8C, 8E, (8F), (8G), (8H)	–17D (99.9–100), 8H (95.7)17D (99.1–100), 8F (82.1–97.9), 8G (77.2–97.4), 8H (94.0–96.8)17D (94.5–100), 8F (82.1), 8G (95.5–95.4), 8H (94.1)
**9/11**§	63	6 of 65 of 6	585	91.98.1	7A, 7B, 9C, 9D, 9E, 11F7A, 7B, 9C, 9D, 9E	2B (43.6–85.1), 3B (49.2–55.0), 6A (23.3–57.4), 8A (22.6–24.2), 9F (23.0–45.4)6A (13.1–58.4), 7C (32.6), 11F (97.8–97.9)
**10**	10	4 of 43 of 42 of 4	811	801010	10A, 10B, 10C, 10D10A, 10C, 10D10A, 10B	–No *cps10B* gene10C (92.0), 10D (93.1)
**12**	28	3 of 32 of 3	199	67.932.1	12A, 12B, 12C(12A), (12B), (12C)	–12A (94.5–97.4), 12B (53.2–92.7), 12C (91.6)
**13**	17	5 of 5	17	100	2A, 2B, 13C, 13D, 13E	6A (21.9–60.8), 12B (62.7)
**14**	5	9 of 98 of 9	41	8020	14A, 14B1, 14B2, 14B3, 14 C-G14A, 14B1, 14B2, 14 C-G	–No *cps14B3* gene
**15**	4	3 of 3	4	100	15A, 15B, 15C	–
**16**	5	6 of 64 of 6	41	8020	16A, 16B, 16C, 16D, 16E, 16F16B, 16C, 16D, 16F	–No *cps16A* and *cps16E* genes
**17**	29	6 of 64 of 63 of 6	8201	27.674.13.4	17A, 2B, 8C, 17D, 17E, 17F2B, 17D, 17E, 17F17D, 17E, 17F	–17A (37.7–65.3), 8A (99.3–99.8), 7C (22.8–53.1)17A (37.7), 8A (99.3), 3B (99.1), 3C (99.3)
**18**	7	3 of 32 of 3	61	85.714.3	18A, 18B, 18C18B, 18C	–18A (50.3), 14A (74.4)
**19**	5	4 of 43 of 42 of 4	311	602020	19A, 19B, 19C, 19D19B, 19C, 19D19C, 19D	–4A (97.2), 19A (23.2)4A (96.6), 19A (13.4), 19B (97.9)
**K2:O7**	23	7 of 76 of 75 of 7	3614	13.026.160.9	8A, 2B, 8C, 2D, 2E, 2F, 2G8A, 2B, 2D, 2E, 2F, 2G8A, 2B, 2D, 2E, 2F	–17A (37.1—45.5), 8C (95.6–97.4)17A (37.7–63.8), 2C (68.9–99.9), 6C (61.4–94.6), 2G (59.7–65.4)
**NT**	8	–	–	–	No match to any profile	–

*The isolates of a serovar are grouped according to the number of *cps* genes detected that match those found in the serovar reference strain. If more than 50% of the genes of the serovar reference strain are found, the isolate is allocated to this serovar.

†The *cps* genes found in the different groups. A gene is regarded as a match provided there is a similarity over 98% and 98% coverage. As some *cps* genes are identical in certain serovars, the *cps* gene is given the name of the serovar with the lowest number. If the gene is not found in all isolates in a group, the gene is put in parenthesis.

‡Other genes are genes found with similarity below the 98% cut-off level or the presence of genes found that differ from the composition found in the serovar reference strain. The pipeline output shows both the similarity and coverage of the different *cps* genes, but for simplicity, the table only shows the similarities.

§In total, 58 isolates (including the type strain of serovar 9) were predicted to be serovar 11. Five isolates had a *cpsF* gene with a similarity in the range 97.8–97.9 to *cps11F* and were predicted to be serovar 9/11. In this table, all 63 isolates are given the designation serovar 9/11.

nt, non-typeable.

Serovar detector allocated 23 isolates to the composite serovar K2:O7 defined by having *cps* genes similar to both serovar 8 and serovar 2 [3]. If a separate profile had not been added for K2:O7, the Serovar detector would have suggested all these strains to be serovar 2.

Serovar detector was unable to separate between serovars 9 and 11. The only distinguishing feature between the *cps* genes of serovars 9 and 11 is the polymorphism in the *cpsF* genes, resulting in an early stop codon for *cps9F*. All typeable isolates, among those that originally were assumed to belong to either serovar 9 or 11, were allocated to serovar 11, including the reference strain for serovar 9. Serovar detector determined isolates to be non-typeable where the *cpsF* gene had an identity and coverage below 98% and could not be allocated to either serovar 9 or 11. These isolates were consequently typed as serovar 9/11. This included five Spanish isolates which had a similarity below 98% to the *cpsF* gene and are listed with a gene frequency of ‘5 of 6’ in Table 2. The five Spanish isolates were closely related to the other serovar 11 or 9/11 strains by SNP analysis. This group of isolates will be referred to as serovar 9/11 in the rest of this manuscript.

There was considerable heterogeneity in the *cpsF* genes among the serovar 9/11 isolates, with several polymorphisms in addition to those found in the serovars 9 and 11 reference strains. The difference in the genomic region of the *cpsF* gene in the reference strains of serovars 9 and 11 is a single deletion of adenosine at position 191 in the *cps11F* gene, resulting in different stop codons (the ORF for *cps9F* is 1,146 bp compared to that of *cps11F* being 1,242 bp long). Among the 63 isolates investigated that supposedly should belong to serovar 9 or 11, only the reference strain of serovar 9 had the predicted ORF of 1,146 bp, although 18 isolates had an A in position 191 similar to serovar 9. Furthermore, 34 isolates had an ORF of 1,242 bp, and 11 isolates had an ORF of 1,182 bp, whereas the remaining 19 isolates had predicted ORFs in the range between 201 and 903 bp (data not shown).

If we disregard the K2:O7 group (23 isolates) and the 9/11 group, only 8 isolates (1.1%) could not be allocated to a serovar by the Serovar detector, as in these 8 isolates <50% of the *cps* genes had >98% similarity to the *cps* genes of one of the serovar reference strains.

### Comparison to PCR

For the 169 Spanish isolates, a close correspondence between the results from the Serovar detector and PCR-based serotyping was found. For 11 strains, the PCR predicted a strain to be serovar 2, while the Serovar detector determined these to be the composite serovar K2:O7. Three Spanish isolates were typed as serovar 6 by both methods, although an ID% and coverage% below 98% were found for *cps6A* and *cps2B* (the *cpsB* gene in the serovar 6 reference strain). One Spanish isolate was typed as serovar 8 by both methods but showed low ID% to *cps8B* in the Serovar detector output. One isolate was non-typeable by both methods.

Among the Spanish isolates, the PCR test allocated 55 isolates to serovar 9/11, as the PCR test was not designed to discriminate these serovars. Among the 55 isolates, 50 were typed by the Serovar detector to be serovar 11 and 5 isolates as serovar 9/11. One isolate, which was non-typeable by PCR, was also allocated to serovar 11 by Serovar detector.

Twenty Spanish isolates were typed as serovar 17 by both methods, although only four out of six *cps* genes were full matches to the serovar reference strain.

In summary, there was a 93% concordance (157/169 isolates) between results from PCR and Serovar detector, with discrepancies due to 11 K2:O7 isolates and 1 9/11 isolate.

### Comparison to serotyping by slide agglutination

In 2021, 119 Danish strains of *A. pleuropneumoniae* were serotyped both by WGS and slide agglutination. For 114 of the strains, the same serovar was determined by both methods. Two non-typeable strains (due to spontaneous agglutination) were, through WGS serotyping, determined to be serovars 6 and 7, respectively. Three strains were typed as serovars 6, 7 and 13 by agglutination, whereas the Serovar detector determined these to be serovars 12, 6 and 18, respectively. Serovar detector results were confirmed through manual investigation of the sequences using CLC. Interestingly, serovar 18 has been isolated in Denmark [8], whereas serovar 13 has never been reported.

### Comparison to SNP grouping

**Fig. 1.**
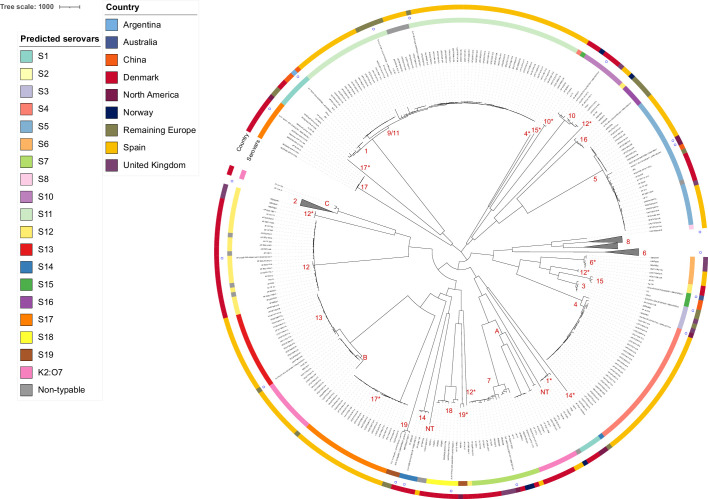
Phylogenetic tree of 732 isolates of *A. pleuropneumoniae*. Isolates containing the reference strains of serovars 2 (*n*=199) and 6 (*n*=60) were collapsed each in one cluster, and serovar 8 was collapsed in two clusters (*n*=145 and 32). The allocation of serovars to the isolates is shown in red numbers 1–19. The cluster marked 9/11 contains isolates received as serovar 9 or 11. If isolates of a serovar are found in groups not containing the serovar reference strain, the groups are marked with an asterisk. Three groups of K2:O7 are marked with letters A–C. NT: non-typeable. Inner circle: colours indicate serovar; outer circle: colour indicates geographical origin. Serovar reference strains are indicated with small, round circles.

To further evaluate the usefulness of the Serovar detector for investigating the genomic variation in the entire dataset, a phylogenetic analysis was performed on all 732 isolates (Fig. 1). To better show the SNP groups found in the collection of isolates and to simplify the presentation, the isolates containing the reference strains of serovars 2 and 6 were collapsed each in one cluster, and serovar 8 was collapsed in two clusters. With few exceptions, all isolates of a serovar belonged to the same phylogenetic group as the serovar reference strains. Isolates of a serovar that were found in clusters distinct from those containing the reference strains are indicated with an asterisk.

Eight strains of serovar 1 clustered close to serovars 9 and 11. In addition, five isolates earlier described as K1:O7 were found in a distinct cluster (1*).

All 199 isolates of serovar 2 clustered together and showed limited genetic variation.

Isolates allocated to the composite serovar K2:O7 were found in three distinct phylogenetic positions (marked A–C in Fig. 1). Nine isolates clustered together on three neighbouring branches (A), all having two *cps* genes similar to serovar 8 (*cps8A* and *cps8C*) and five genes similar to serovar 2 (*cps2B* and *cps2D–G*), corresponding to the description of K2:O7 in Bossé *et al*. [3,8]. These isolates clustered close to the serovar 7 isolates in the phylogenetic analysis. A second group consisted of 12 isolates originating from Spain and France (B), all having identity and coverage below 65% to the *cps8C* and the *cps2G* genes, which were located close to the serovar 13 cluster. Finally, two isolates clustered close to the serovar 2 group (C). These strains had higher homology to *cps17A* than to *cps8A* and had an identity and coverage around 45% to the *cps8C* gene. If the pipeline had not included a separate profile for K2:O7, all 23 isolates would have been allocated to serovar 2, meaning that the three groups A–C would have been indicated as 2* in Fig. 1.

Isolates allocated to serovars 3 and 4 were found in two distinct clusters, with the exception of a single Spanish serovar 4 isolate, which was found on a distinct branch (4*).

Serovar 5 was found in two closely related clusters, one of the clusters containing all isolates from Spain. One non-typable strain was also found in this group, and as *cps5A* and *cps5C* had similarities between 95 and 97% to the serovar reference strain, it was not allocated to the serovar by Serovar detector. This isolate had been typed as serovar 5 by PCR.

Sixty of the isolates allocated to serovar 6 were found in a genetically homogeneous cluster, whereas six isolates from the UK and Spain were located separately (6*). These six isolates contained five out of seven of the *cps* genes found in the serovar reference strain, and the last two genes had an identity and coverage below 98% to *cps8A* and *cps8B* (=*cps2B*).

The 15 isolates of serovar 7 were found in one cluster located close to the K2:O7-A group.

Serovar 8 strains were divided into two distinct but affiliated clusters containing isolates from the UK and Norway. Danish strains were found in both these groups. This corresponds to that earlier reported by Cohen *et al*. [26]. In addition, a single Spanish isolate was located on a neighbouring branch.

Serovars 9 and 11 were found in a single cluster with limited variation close to the isolates of serovar 1. There was no clear separation in the SNP analysis between isolates received as serovar 9 or 11 or the group of Spanish isolates that had been allocated to serovar 9/11 by PCR. In addition, five Spanish isolates that were non-typeable by PCR were found in this same cluster.

Eight isolates of serovar 10 were located on two closely affiliated clusters whereas two Danish isolates were found in a more deeply branching cluster (10*). These isolates correspond to a group of serovar 10 isolates that earlier were found to be genetically distinct by amplified fragment length polymorphism (AFLP) typing [10].

Serovar 12 was found to be genetically quite heterogeneous. Most Danish serovar 12 strains were found in the same cluster together with four non-typeable isolates. These four isolates had all the serovar 12-specific *cps* genes, but <50% were full matches as required for typing by Serovar detector. Three serovar 12 isolates from the UK were found in a separate cluster positioned between serovars 2 and 12. In addition, three isolates allocated to serovar 12 by Serovar detector were found in three distinct and deeply branching leaves (12*) in the tree.

All isolates of serovars 13 and 16 were found in separate, distinct clusters.

Isolates of serovar 14 from Denmark were all found in a distinct cluster, while a single isolate from Estonia was located in a remote position on a separate branch (14*).

Serovar 15 was found as a distinct group; however, a single Danish isolate was located on a separate branch (15*).

Serovar 17 was divided into three separate clusters containing isolates from Denmark and Spain. Two clusters, one of which contained the serovar reference strain, were found at a position deeply branching from serovars 1/9/11. A group of Spanish strains was found in a remotely positioned and separate cluster (17*).

Serovar 18 was only represented by Danish isolates and was a distinct cluster. One non-typeable isolate had *cps* genes with <98% homology to the *cps* gene of the serovar reference strain.

Serovar 19 was found in two separate clusters, one containing the Danish serovar reference strain together with two isolates from Switzerland, while the other cluster (19*) contained strains from Canada and Denmark.

Finally, two non-typeable isolates were included in the analysis, which showed no close genetic affiliation to any of the other isolates, as the *cps* genes only had low homology to any group. These two isolates (NT) were located on two separate branches in the SNP analysis.

## Discussion

### Performance of Serovar detector

Serovar detector allocated 696 of the 732 isolates (95.1%) to one of the 19 recognized serovars. In addition, 23 were determined to be the composite serovar K2:O7, while 5 isolates were allocated to serovar 9/11. In total, only eight of all isolates (1.1%) could not be allocated to any of the earlier described groups by the method.

Serovar detector was unable to separate between highly similar sequences. We believe this to be an attribute of the Kmer-aligner ConClave scoring, where the best mapping template sequences must be accepted in the best matching query sequences. As described earlier, the *cps9F* gene sequence contains a stop codon earlier than *cps11F*. This, combined with the ConClave scoring scheme, would result in reads mapping better to *cps11F* than *cps9F*, which likely explains the absence of *cps9F* and why the Serovar detector was not able to separate between serovars 9 and 11.

For some serovars, not all expected genes were detected, for instance within serovar 8, where 137 out of 172 isolates only contained seven out of the expected eight *cps* genes. In all these strains, *cps17D* was detected instead of *cps8D*, two genes differing by only two SNPs. This is likely also an attribute of the Kmer-aligner ConClave scoring scheme, where the best mapping template is accepted.

Even despite the issues with distinguishing similar genes, Serovar detector was shown to be a robust and precise method for determining serovars. Furthermore, the generic approach of the Serovar detector would enable the methodology to be applied for other bacterial species, given that a suitable reference database alongside a list of gene profiles defining the different groups would be provided.

Another method for WGS-based *in silico* serotyping of *A. pleuropneumoniae* was recently published by Li *et al*. [34]. Here the entire region between the *modF* and *ydeN* genes was used for prediction and not only the *cps* genes. In addition to the serovar reference strains, only 97 genome assemblies were tested, representing 13 of the serovars. This method also offers an opportunity for the manual inspection of the results and a full list of identities and missing genes. This method does not, however, use a cut-off limit for assigning a gene to a serovar, and the authors state that capsular locus types will be assigned even with extremely low identities. Surprisingly, they claim that the method will be able to distinguish serovars 9 and 11 based on the investigation of two field strains.

### WGS compared to PCR

A close correspondence between the results from the Serovar detector and PCR-based serotyping was observed among the 169 Spanish strains [17]. For 11 strains, the PCR predicted a strain to be serovar 2, whereas the Serovar detector determined the type to be K2:O7. This is as expected since the PCR test was designed to type isolates of K2:O7 as serovar 2, as it targets the common *cps2E* gene [3].

### Genetic lineages within serovars

The SNP typing was performed to assess whether the serovars determined by the Serovar detector represent genetically homogeneous groups. Disregarding the isolates of K2:O7 (*n*=23), 9/11 (*n*=63) and the 8 non-typeable isolates, 93% of the remaining 640 isolates clustered together with the respective serovar reference strains. Divergent genetic groups were found in serovars 1, 4, 6, 12, 14, 15, 17 and 19.

In serovar 1, a distinct SNP cluster contained five isolates earlier designated K1:O7 [9]; the genetic uniqueness of this has earlier been shown by AFLP typing [10]. In serovar 4, a single isolate from Estonia was found on a separate branch. All isolates of serovar 6 were found in a homogeneous cluster; however, six isolates were found on a separate branch. The 28 isolates of serovar 12 were found in five distinct clusters. The majority of the isolates clustered together with the serovar reference strain; however, seven isolates were located on four distinct branches in the tree.

In both serovars 14 and 15, a single isolate was found on distinct branches. Finally, the five isolates of serovar 19 were found in two distinct groups. The genetic heterogeneity of this group was also indicated by Stringer *et al*. [4] as the Danish serovar reference strain and a Canadian strain were reported to have different O-antigens (K19:O3 and K19:O4, respectively).

Among the eight isolates designated as non-typeable by Serovar detector, six were found in the clusters of the serovar reference strains of serovars 5, 12 (*n*=4) and 18, respectively. All six isolates contained the expected *cps* genes of these serovars but at a similarity below 98%, thus indicating that the designation non-typeable in these cases might be dependent on sequencing quality. Finally, two non-typeable isolates were found as single isolates on distinct branches in the tree.

### Discrimination between serovars 9 and 11

Serovar detector was unable to distinguish between isolates of serovars 9 and 11. Identifying these serovars has represented a challenge by all available serologic and genetic methods, as the serovars have almost identical capsule and O-antigens as well as virulence factors (i.e. Apx toxins). The SNP analysis could not give a clear distinction between the serovar reference strains of serovars 9 and 11, and all investigated isolates were located in a highly affiliated cluster. This included strains originally received as serovar 9 or 11, as well as a group of 56 Spanish isolates that had been typed as serovar 9/11 by PCR [17]. A publication has recently addressed the challenge of developing a test to differentiate between serovars 9 and 11 [35]. This investigation included the partial or complete sequencing of the *cpsF* gene in 134 isolates and genomic analysis of another 19 isolates by WGS. This investigation indicates that the main genetic variation among isolates allocated to serovars 9 and 11 is a number of point mutations in the *cpsF* gene, some of which introduce new reading frames and shorter transcriptomes compared to the OPF of 1,242 bp found in serovar 11. The ORF of 1,146 bp found in the serovar 9 reference strain was unique in the collection. The investigation hypothesizes that all OPFs shorter than 1,246 bp would lead to a non-functional *cpsF* gene, resulting in a capsular structure similar to that found in the serovar 9 reference strain. This genetic heterogeneity also explains why it is impossible to design a PCR test for separation of these two serovars, and the authors conclude that for most practical purposes it will be a valid approach to characterize this group of isolates as the hybrid serotype 9/11. A precise identification of serovars 9 and 11 could in principle be obtained by WGS provided all variants of the *cpsF* gene could be allocated to one of the serovars. In the present investigation, we found that 11 isolates had a predicted *cpsF* ORF of 1,182 bp, and it is not possible based on Arnal Bernal *et al*. [35] to unequivocally conclude which capsular phenotype this would give rise to. In this situation, we find that a practical solution is to just refer to this group of isolates as serovar 9/11.

The methodology used in the Serovar detector is designed to detect and interpret the composition of capsular genes and not to detect and interpret single polymorphisms. Detecting polymorphisms would require the development of a separate module, which is outside the scope of Serovar detector.

### Identification of serovar K2:O7

Strains with an unusual pattern of surface antigens designated K2:O7 were described by Nielsen *et al*. [7], characterized by having a CPS similar to that of serovar 2 and a lipopolysaccharide structure similar to that of serovar 7. Isolates of this group were further characterized by Bossé *et al*. [3], who described that the capsular operon consisted of *cps* genes similar to serovars 2 and 8. Some of the isolates of this group were found to possess unique compositions of *cps* genes and were allocated to the new serovars 17 and 18 [8]. The present investigation shows that this group is genetically heterogeneous and might deserve further study. Inclusion of this group in the validation of the Serovar detector shows the value of including additional groups to investigate strains showing divergent properties. Isolates within this group might, for instance, have virulence properties that might make it relevant to separate them from isolates of serovar 2, for instance, by only expressing ApxII and not ApxIII [8,12].

To *et al*. [12] recently investigated 12 isolates previously designated as serovar 4 or non-typeable. One isolate was found to be K2:O7 and 11 isolates had a truncated *cps2G gene* and O4 O-antigen. Interestingly, we also found that 14 isolates of the K2:O7 group had a similar low similarity and coverage of the *cps2G* gene (Table 2). This also underlines the variability found in the *cps* operon and, at the same time, the necessity to be cautious before drawing conclusions based on nucleotide differences in single genes and that thorough investigations should be performed before introducing nomenclatural changes and defining new serovars.

An example of this is the recent publication of serovar 6b:O3 [13]. Here, a variant of the serotype 6 locus was published with low similarity to the *cpsA-C* genes found in the serovar reference strain. As seen from Table 2, a low similarity in these genes was also observed for strains in our collection. We question whether the addition of letters to the serovar designation represents a practical and sustainable way of describing future variations within serovars of *A. pleuropneumoniae*.

## Conclusions

This investigation has shown that WGS-based serotyping is a robust method for allocating unknown isolates to a serovar. In the present investigation, isolates from 18 countries were included, representing all published serovars as well as additional aberrant groups that might represent a challenge to a typing method. The method gives quick and exact serotyping in addition to supplying information on variation in the detected *cps* genes that might give rise to further investigations and descriptions of additional serovars. The variation existing in the *cps* genes also indicates that caution is required when relying only on sequence variation within this operon when characterizing new groups within the species. The relevance of such variation should be substantiated by other factors that can support the importance of such groups, e.g. prevalence of the varieties, distinct serological reaction in pigs, the presence of virulence genes encoding Apx toxins, O-antigens and genetic distinctness as shown by WGS. The phylogenetic investigation showed that although most of the isolates of a certain serovar are genetically homogeneous, there exists additional genetic variation that cannot easily be explained by the *cps* genes alone. This indicates that SNP-based phylogeny or wgMLST might be valuable supplements for assessing the variation of *A. pleuropneumoniae* in a given region. A possibility might be to supplement the serovar designation with a genetic affiliation of an isolate, e.g. by determining a core genome MLST scheme.

In conclusion, the WGS serotyping performed by Serovar detector has been demonstrated both to be robust and to correlate well with classical serotyping methods, and it has already been successfully implemented for serotyping of isolates in a diagnostic setting. The availability of WGS data facilitates further characterization of isolates, for instance, by screening for resistance and virulence determinants and investigating the clonality of epidemiologically related isolates.

## Supplementary material

10.1099/mgen.0.001434 Uncited Table S1.

10.1099/mgen.0.001434 Uncited Table S2.

## Abbreviations

AFLP: amplified fragment length polymorphism
CPS: capsular polysaccharide
WGS: whole genome sequencing
