# Supplementary material for: Serotyping of Actinobacillus pleuropneumoniae based on whole genome sequencing: validation of a bioinformatic tool
Source: Microb Genom. 2025 Jul 15;11(7):001434. doi: 10.1099/mgen.0.001434 (PMC12263286; doi:10.1099/mgen.0.001434)
Supplement: Uncited Table S2. [file mgen-11-01434-s002.pdf]

Table S1. *Actinobacillus pleuropneumoniae* serovar *cps* profiles

Serovar	Reference strain	Capsular type[a]	*cps* genes
1	4074	2	1A, 1B, 1C, 1D
2	S1536	1	2A, 2B, 2C, 2D, 2E, 2F, 2G
3	JL03	1	3A, 3B, 3C, 3D, 3E, 3F, 3G
4	M62	2	4A, 4B, 4C
5	L20	3	5A, 5B, 5C
6	Femø	1	6A, **2B,** 6C, 6D, 6E, 6F, 6G
7	AP76	1	7A, 7B, 7C, 7D, 7E
8	MIDG2331	1	8A, **2B**, 8C, 8D, 8E, 8F, 8G, 8H
9	CVJ13261	1	**7A, 7B,** 9C, 9D, 9E, 9F
10	D13039	3	10A, 10B, 10C, 10D
11	56153	1	**7A, 7B, 9C, 9D, 9E,** 11F
12	1096	2	12A, 12B, 12C
13	MIDG2225	1	**2A, 2B,** 13C, 13D, 13E
14	MIDG2226	2	14A, 14B1, 14B2, 14B3, 14C, 14D, 14E, 14F, 14G
15	HS143	2	15A, 15B, 15C
16	MIDG3609	4	16A, 16B, 16C, 16D, 16E, 16F
17	MIDG3419	1	17A, **2B, 8C,** 17D, 17E, 17F
18	MIDG3434	2	18A, 18B, 18C
19	7213384	2	19A, 19B, 19C, 19D
K2:O7[a]	S1536 & MIDG2331	1	**8A, 2B, 8C, 2D, 2E, 2F, 2G**

[a]: Bossé et al., 2018. This publication also includes a figure visualizing the genes included in the different *cps*-loci of the serovars: https://ars.els-cdn.com/content/image/1-s2.0-S0378113518303626-gr1.jpg

Some *cps* genes are identical in certain serovars, the *cps* gene is given the name of the serovar with the lowest number. These genes are shown in bold.

Supplementary Table S2. Similarities between *cps* genes of *Actinobacillus pleuropneumoniae* serovars.

Capsule type 1

Serovar	Capsular type	*cps* -genes
2	1	2A, 2B, 2C, 2D, 2E, 2F, 2G
3	1	3A, 3B, 3C, 3D, 3E, 3F, 3G
6	1	6A, 2B, 6C, 6D, 6E, 6F, 6G
7	1	7A, 7B, 7C, 7D, 7E
8	1	8A, 2B, 8C, 8D, 8E, 8F, 8G, 8H
9	1	7A, 7B, 9C, 9D, 9E, 9F
11	1	7A, 7B, 9C, 9D, 9E, 11F
13	1	2A, 2B, 13C, 13D, 13E
17	1	17A, 2B, 8C, 17D, 17E, 17F

cpsA-genes

	2	3	6	7	8	9	11	13
3	99.825							
6	97.725	97.725						
7	94.066	94.066	95.455					
8	88.482	88.842	90.035	94.401				
9	94.066	94.066	95.455	100	94.401			
11	94.066	94.066	95.455	100	94.401	100		
13	100	99.825	97.725	94.066	88.482	94.066	94.066	
17	92.745	92.745	94.318	95.188	95.538	95.188	95.188	92.745

cpsB-genes

	2	3	6	7	8	9	11	13
3	94.639							
6	100	94.639						
7	99.767	94.872	99.767					
8	100	94.406	100	99.700				
9	99.767	94.872	99.767	100	99.767			
11	99.767	94.872	99.767	100	99.767	100		
13	100	94.639	100	99.767	100	99.767	99.767	
17	100	94.406	100	99.700	100	99.767	99.767	100

cpsC-genes

	2	3	6	7	8	9	11	13
3	95.137							
6	98.946	95.811						
7	91.137	95.157	90.765					
8	90.133	91.266	89.939	94.316				
9	89.516	95.157	89.232	95.098	92.851			
11	89.516	95.157	89.232	95.098	92.851	100		
13	97.898	96.436	97.806	92.416	88.026	88.410	88.410	
17	90.133	91.266	89.939	94.316	100	92.851	92.851	88.026

cpsD-genes

	2	3	6	7	8	9	11	13
3								
6								
7	80.663							
8			96.653					
9		84.399						
11		84.399				100		
13								
17			96.721		99.932			

cpsE-genes

	2	3	6	7	8	9	11	13
3								
6								
7								
8			85.659					
9								
11						100		
13								
17					99.710			

Other Type 1 cps genes with homologies

6G - 8G	90.317
6G - 17G	85.812
6G - 8H	90.318
6G - 17F	89.426
8H - 19F	94.231
9F - 11F	99.909

Capsular type 2

Serovar	Capsular type	*cps* -genes
1	2	1A, 1B, 1C, 1D
4	2	4A, 4B, 4C, 4D
12	2	12A, 12B, 12C
14	2	14A, 14B1, 14B2, 14B3, 14C, 14D, 14E, 14F,
15	2	15A, 15B, 15C
18	2	18A, 18B, 18C
19	2	19A, 19B, 19C, 19D

cpsA-genes

	1	4	12	14	15	18
4	99.281					
12	95.619	95.524				
14	99.641	99.281	95.714			
15	-	-	-	-		
18	99.102	98.742	95.619	99.461	-	
19	99.461	99.461	95.905	99.820	-	99.281

Footnote: The table shows similarities observed between *cps* genes of certain serovars of *Actinobacillus pleuropneumoniae* capsular types 1 and 2 (Bossé et al., 2018). Genes with identical sequences are marked with pink.
